# Different Placebos, Different Mechanisms, Different Outcomes: Lessons for Clinical Trials

**DOI:** 10.1371/journal.pone.0140967

**Published:** 2015-11-04

**Authors:** Fabrizio Benedetti, Sara Dogue

**Affiliations:** 1 University of Turin Medical School, Neuroscience Department, Turin, Italy; 2 Plateau Rosa Labs, Breuil-Cervinia, Italy, Zermatt, Switzerland; The James Cook University Hospital, UNITED KINGDOM

## Abstract

Clinical trials use placebos with the assumption that they are inert, thus all placebos are considered to be equal. Here we show that this assumption is wrong and that different placebo procedures are associated to different therapeutic rituals which, in turn, trigger different mechanisms and produce different therapeutic outcomes. We studied high altitude, or hypobaric hypoxia, headache, in which two different placebos were administered. The first was placebo oxygen inhaled through a mask, whereas the second was placebo aspirin swallowed with a pill. Both placebos were given after a conditioning procedure, whereby either real oxygen or real aspirin was administered for three consecutive sessions to reduce headache pain. We found that after real oxygen conditioning, placebo oxygen induced pain relief along with a reduction in ventilation, blood alkalosis and salivary prostaglandin (PG)E2, yet without any increase in blood oxygen saturation (SO2). By contrast, after real aspirin conditioning, placebo aspirin induced pain relief through the inhibition of all the products of cyclooxygenase, that is, PGD2, PGE2, PGF2, PGI2, thromboxane (TX)A2, without affecting ventilation and blood alkalosis. Therefore, two different placebos, associated to two different therapeutic rituals, used two different pathways to reduce headache pain. The analgesic effect following placebo oxygen was superior to placebo aspirin. These findings show that different placebos may use different mechanisms to reduce high altitude headache, depending on the therapeutic ritual and the route of administration. In clinical trials, placebos and outcome measures should be selected very carefully in order not to incur in wrong interpretations.

## Introduction

The use of placebo control groups is crucial in the current methodology of clinical trials. However, whereas a placebo is usually defined as an inert treatment devoid of any intrinsic therapeutic property, there is today some evidence that all placebos are not equal, some of them being more effective than others, for example in migraine [1] and osteoarthritis [2]. The differences across different placebos, e.g. oral, subcutaneous, topical, intra-articular placebos, as well as sham acupuncture needles, indicate that placebos are not inert but rather they are made of many psychosocial elements that build up the ritual of the therapeutic act [3,4,5].

Recently, high altitude, or hypobaric hypoxia, headache has emerged as an interesting model to better understand the biological underpinnings of placebo analgesia [6,7]. This kind of headache is part of a clinical condition known as acute mountain sickness (AMS), which is usually diagnosed by means of the Lake Louise Score (LLS) questionnaire [8]. This is aimed at detecting several symptoms, such as headache, nausea/vomiting, dizziness, insomnia, as well as neurological symptoms, which emphasize the complex nature of this hypoxia-related clinical syndrome. AMS is triggered by the drop in atmospheric oxygen pressure at high altitude [9,10].

**Fig 1:**
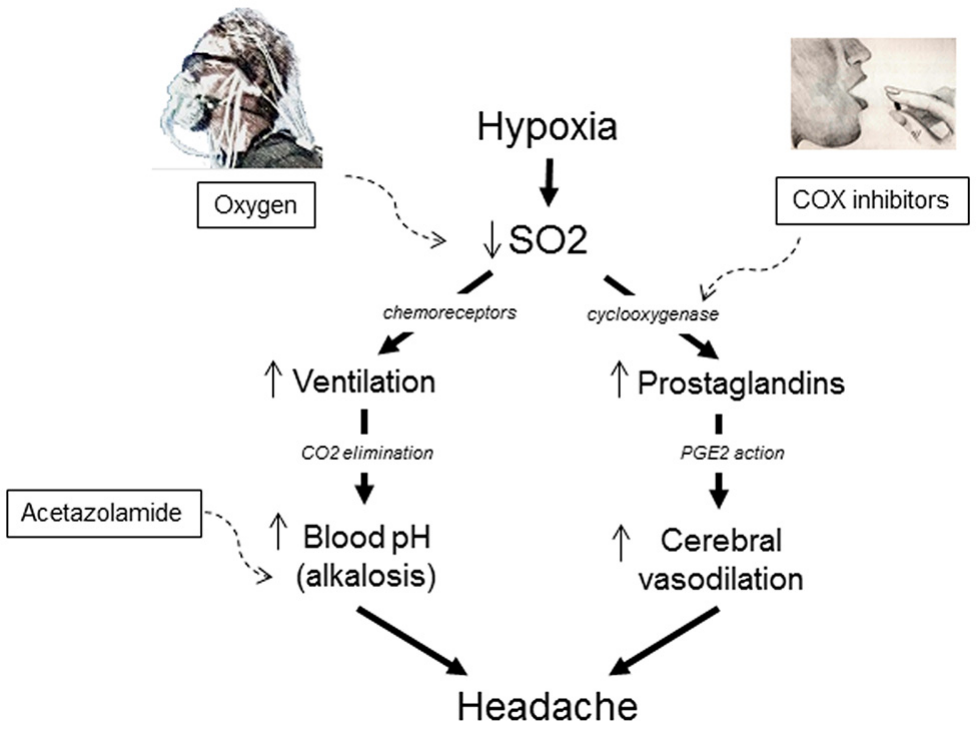
Rationale of the study. High altitude headache can be due to at least two pathways. The first is the reduction of blood oxygen saturation (SO2), which triggers compensatory hyperventilation and excessive elimination of carbon dioxide (CO2) which, in turn, increases blood pH (alkalosis). The importance of alkalosis in high altitude headache is shown by the therapeutic effects of acetazolamide, that reduces blood pH. The second pathway is the activation of cyclooxygenase (COX) by hypoxia with an increase in prostaglandin (PG) synthesis which, in turn, induces cerebral vasodilation. High altitude headache can be treated with oxygen or COX inhibitors, like aspirin, and these treatments require two different therapeutic rituals. In the former, oxygen is breathed through a mask, in the latter, aspirin is a pill that is taken orally. This allows the study of the mechanisms of two different placebos associated to two different rituals.

As depicted in Fig 1, there are at least two pathways triggering high altitude headache. The first is represented by the acute effects of hypoxia on prostaglandin (PG) synthesis through the cyclooxygenase (COX) enzyme, with the formation of PGD2, PGF2, PGE2, PGI2 (prostacyclin), and thromboxane (TX) A2 [7,11]. One of the most important effects of these eicosanoids, particularly PGE2, is represented by vasodilation, which is thought to be the principal factor inducing acute hypoxia headache [12–16], although the direct stimulation of nociceptive afferents may also occur [17]. However, a second pathway that is important in high altitude headache is represented by hypoxia-related hyperventilation that, in turn, induces the excessive elimination of carbon dioxide (CO2) with the consequent increase in blood pH (alkalosis) [18]. To support the important role of alkalosis in AMS and high altitude headache is the therapeutic effect of blood pH reduction by means acetazolamide [19].

Besides the reduction of alkalosis with acetazolamide, there at least two other effective treatments for high altitude headache (Fig 1): oxygen inhalation [6,20] and oral cyclooxygenase inhibitors, such as aspirin [7,21]. Whereas the former restores blood oxygen saturation (SO2), thus decreasing hyperventilation and alkalosis, the latter inhibits the hypoxia-activated cyclooxygenase and PG synthesis, thus reducing cerebral vasodilation.

Interestingly, by observing Fig 1, oxygen therapy and aspirin therapy require two different therapeutic rituals, the first breathing through a mask and the second swallowing a pill. Therefore, they represent an interesting model to understand differences and similarities between placebo oxygen and placebo aspirin. Since previous studies showed that high altitude headache is affected by placebo only after a pre-conditioning procedure [6,7], in the present study we analyzed the placebo response after repeated associations between headache reduction and either the oxygen mask ritual or the aspirin pill ritual.

## Materials and Methods

### Subjects and study location

**Fig 2:**
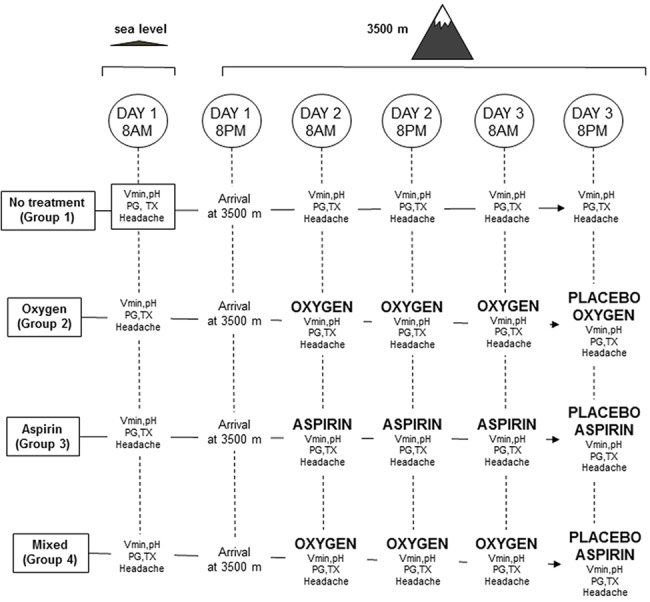
Experimental design. The sequence of test sessions is shown for all groups, starting from 8:00 AM of Day 1 at sea level to 8:00 PM of Day 3 at 3500 m. Vmin = minute ventilation; PG = prostaglandins; TX = thromboxane.

A total of 28 healthy volunteers (see Table 1 for details), were recruited from the student population of the Medical School and Nursing School of the University of Turin and from the School of High Altitude Medicine and Physiology. The participants signed a written informed consent form in which the experimental procedure was described in detail. The study was approved by the ethics committee of the High Altitude School of Medicine and Physiology. All subjects engaged in recreational fitness training and were asked to avoid hard exercise starting from at least 24 h before the experimental sessions. They were either non-smokers or light smokers (less than 10 cigarettes/day). The random distribution of the smokers is shown in Table 1. During the first visit, their medical history was recorded in order to rule out main diseases, and an electrocardiogram was performed to assess their heart response to exercise. The subjects were randomly subdivided into 4 groups (Table 1 and Fig 2). All the subjects were asked to refrain from consuming coffee, tea, or other caffeine-containing beverages for 48 h before the experimental sessions, as well as alcohol and any drug. None of them consumed more than 2 cups of coffee/day, corresponding to less than 100 mg/day of caffeine. The morning experimental sessions were conducted after a standardized breakfast had been consumed. This consisted of a cup of milk or fruit juice and a muffin or biscuits, whose energy content was about 2000 kJ. The afternoon experimental sessions were conducted after a 4000 kJ lunch at 1:00 pm, consisting of pasta, fruit juice and fruit.

**Table 1:** Subject characteristics in the different groups (m = males; s = smokers; BMI = body mass index).

Group	No treatment (Group 1)	Placebo Oxygen (Group 2)	Placebo aspirin (Group 3)	Mixed (Group 4)
Number of subjects	7 (4m, 2s)	7 (5m, 2s)	7 (5m, 1s)	7 (4m, 1s)
Age (years)	24.1 ± 1.44	23.5 ± 1.76	23.3 ± 1.9	23.8 ± 2.18
Weight (kg)	67.6 ± 7.53	69.4 ± 8.45	70.2 ± 8.88	68.9 ± 9.47
BMI (kg/m^2^)	22.03 ± 4.05	21.84 ± 3.19	21.56 ± 3.14	21.66 ± 4.24

The experiments were performed in our laboratories at the Plateau Rosa Research Station in the Matterhorn area at the Italian-Swiss border (Breuil-Cervinia area on the Italian side and Zermatt on the Swiss side) at an altitude of 3500 meters, that can be reached through three cableways in 30 minutes, starting from an altitude of 2050 m in Breuil-Cervinia. At 3500 m air pressure is about 490 mmHg (760 mmHg at sea level) and oxygen pressure about 102 mmHg (159 mmHg at sea level). This corresponds to a blood oxygen saturation (SO2) in the range of 83–90% (98–99% at sea level), depending on different individuals. Ambient temperature inside the Research Station was always maintained at 18°C. All the experiments were performed under acute hypobaric hypoxia, that is, in the first 48 hours after reaching 3500 m.

### Experimental procedure

Physiological parameters were monitored by means of an Equivital EQ02 LifeMonitor (Hidalgo, Cambridge, UK), providing recordings of the electrocardiogram (EKG), blood oxygen saturation (SO2), movements (accelerometer), head and axillary temperature. A wireless connection allowed monitoring through a Samsung Galaxy Tablet. Subjects breathed through a mask connected to a Cosmed K4b2 apparatus (Rome, Italy) for the recording of minute ventilation (Vmin). A small tube in the mask allowed us to take saliva samples at any time. A blood sample from the fingertip was taken for blood pH measurement.

The subjects were randomly subdivided into 4 groups, but males/females balance was taken into account by randomizing first males then females. Gender, age, weight, and body mass index (BMI) are shown in Table 1 for each group. As shown in Fig 2, all groups were first tested at sea level in our labs in Turin (elevation: 240 m) at 8:00 am of Day 1, then they went up to 3500 m at 8 pm of the same day. The first test at high altitude was performed at 8:00 am of Day 2, the second test at 8:00 pm of Day 2, the third test at 8:00 am of Day 3, and the fourth test at high altitude at 8:00 pm of Day 3. On the basis of our previous study, in which headache was worsened by exercise [6], the subjects had to complete 3000 steps on a stepper at a pace of 2 steps/s following a timer. They were not allowed to drink during exercise. Headache pain was assessed 30 min after exercise by means of a NRS ranging from 0 = no pain to 10 = unbearable pain, along with SO2, Vmin, blood pH, and salivary PGD2, PGE2, PGF2, PGI2, TXA2. We assessed post-exercise headache because our previous study showed a substantial effect of placebo on post-exercise headache pain but not on headache pain at rest [6].

Although all this sequence was identical across all 4 groups, the groups differed as follows (Fig 2). 1) The no-treatment group did not undergo any treatment and was used to evaluate the natural course of headache and all the physiological parameters during the 48 hours of the experiment at high altitude. 2) The placebo oxygen group was given real oxygen for 3 consecutive sessions (see Fig 2), then real oxygen was replaced with sham oxygen and the subjects were told that oxygen was being delivered. The experimenter was blind, according to a double-blind protocol. This group was used to assess the effects of placebo oxygen after oxygen conditioning. 3) The placebo aspirin group was given oral aspirin for 3 consecutive sessions, then aspirin was replaced with oral placebo aspirin according to the same double-blind paradigm. This group was used to assess the effects of placebo aspirin after aspirin conditioning. 4) The mixed group was given real oxygen for 3 consecutive sessions, then inhaled oxygen was replaced with oral placebo aspirin. This group was used to see the effects of the response to oral placebo aspirin following pre-conditioning with inhaled oxygen, that is, conditioning with a placebo ritual (oxygen mask) and test with a different placebo ritual (oral pill).

### Data analysis

Saliva samples were collected by means of a syringe. All samples were kept at -20°C until preparation for analysis. PG analysis started by thawing the saliva samples at room temperature and by recording the volume of each sample. Then, the samples were centrifuged at 3000 rpm at 4°C and the supernatant utilized for PG and total protein analysis. We determined all the main products of cyclooxygenase, the enzyme which transforms arachidonic acid into PGH2 which, in turn, is transformed into PGD2, PGE2, PGF2, PGI2, TXA2. By using ELISA kits (Cayman Chemical, Ann Arbor, MI), we analyzed PGD2, PGE2, and 8-isoprostane PGF2a (PGF2) directly, whereas PGI2 (prostacyclin) was analyzed by assessing its stable metabolite 6-keto PGF1a, and TXA2 assessed through its stable metabolite TXB2. In order to control for artifact variance in the ELISA assay, the amount of PG was normalized to the volume of saliva collected and amount of total protein, which was determined using a standard protein assay (Bio-Rad Laboratories, Hercules, CA).

Minute ventilation (Vmin) was expressed according to standard measurement, that is, as the product of respiratory rate and Tidal volume. Blood pH was measured by means of the glass electrode method, and the Rosenthal correction factor was calculated according to the temperature recorded. The correction factor corresponds to a change in pH of 0.015 pH units per degree centigrade change in temperature [22].

### Statistical analysis

A first within-group analysis was performed by means of repeated measures ANOVA followed by the Student-Newman-Keuls (SNK) post-hoc test for multiple comparisons, in order to see the changes within a single group. Before performing the ANOVA, we used the Mauchly’s sphericity test to verify that the variances of the differences between all possible pairs of groups were equal. In no case sphericity was violated. Effect sizes were calculated by means of Cohen’s *d*. Then, we performed a between-groups analysis by computing the differences of the means and the 95% confidence intervals (CI). The effect of placebo oxygen is represented by the difference between the placebo oxygen (Group 2) and no-treatment group (Group 1). Similarly, the effect of placebo aspirin is represented by the difference between the placebo aspirin (Group 3) and the no-treatment group (Group 1). The effect of placebo aspirin after oxygen conditioning is represented by the difference between Group 4 and Group 1. In addition, differences in placebo responses after placebo oxygen and placebo aspirin were assessed by comparing Groups 2 and 3. The raw data for each group are shown in S1 Table.

## Results

### Within-group analysis

**Fig 3:**
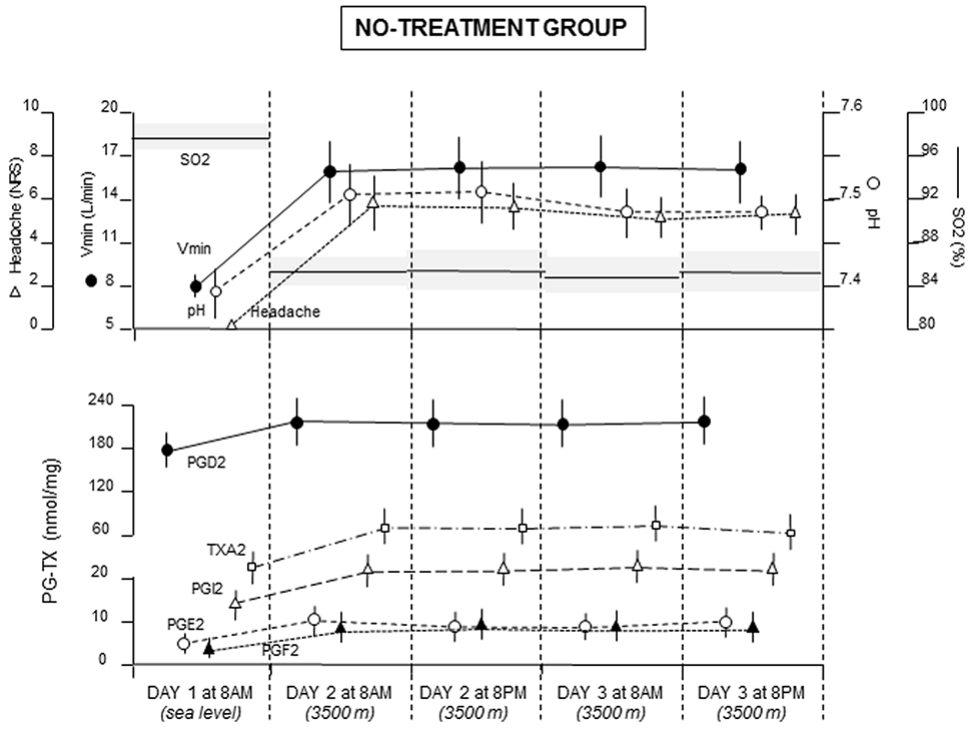
No-treatment group. Natural course of SO2, Vmin, pH, headache pain, PGD2, PGE2, PGF2, PGI2, TXA2 (means+SD) for the first 48 h after arrival at 3500 m, showing the stable conditions of all these physiological parameters at high altitude.

A first intra-group analysis showed a decrease at high altitude compared to sea level in the no treatment group for SO2 (F(4,24) = 108.014, P<0.0001, by ANOVA), and increases in Vmin (F(4,24) = 3.769, P<0.02), pH (F(4,24) = 11.304, P<0.0001) headache (F(4,24) = 79.219, P<0.0001), PGD2 (F(4,24) = 14.438, P<0.0001) PGE2 (F(4,24) = 2.87, P<0.05), PGF2 (F(4,24) = 3.824, P<0.02), PGI2 (F(4,24) = 4.132, P<0.015), TXA2 (F(4,24) = 12.472, P<0.0001). We found no significant changes across the 4 sessions at high altitude (Fig 3), thus showing that all these parameters were stable over time.

**Fig 4:**
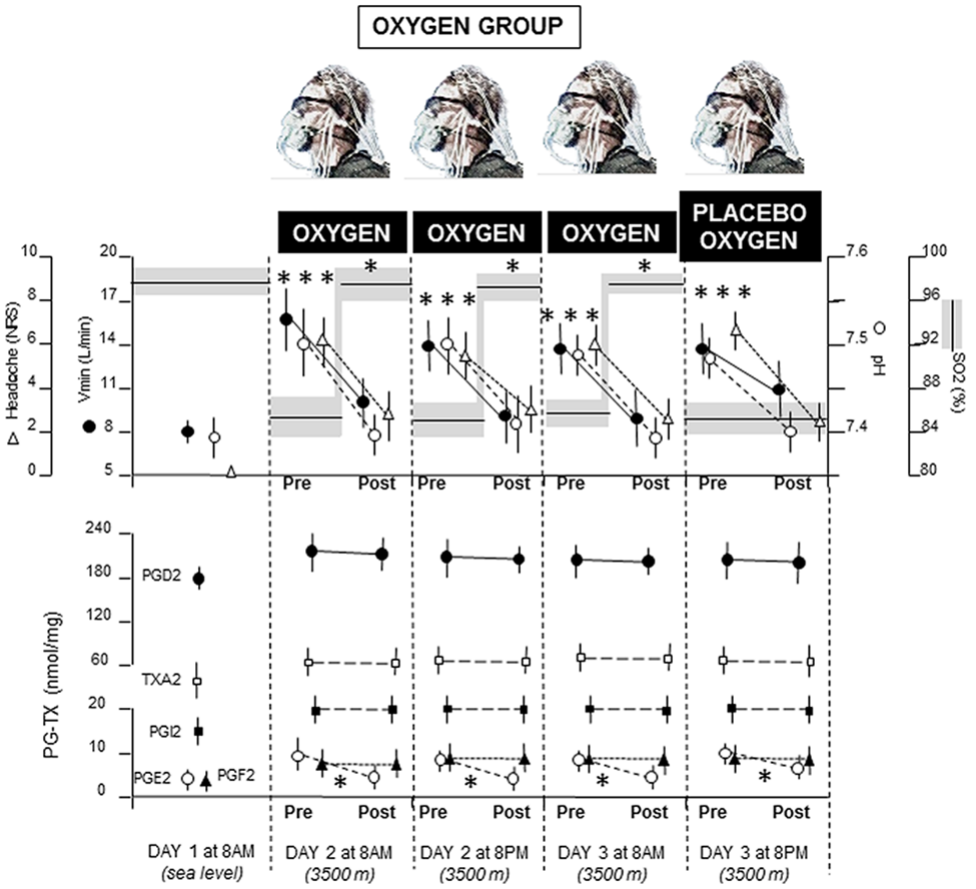
Means (+SD) for all the measurements in the oxygen group. After oxygen administration for three consecutive sessions, real oxygen was replaced with placebo oxygen. Note that placebo mimicked the effects of real oxygen for headache pain, Vmin, pH and PGE2, but it had no effect on SO2, PGD2, PGF2, PGI2, TXA2. *P<0.01.

Similarly, in the oxygen group (Fig 4), SO2 decreased at high altitude whereas all other parameters increased. Oxygen administration on day 2 and 3 increased SO2 (P<0.01 by SNK test), along with a decrease in Vmin (P<0.01), pH (P<0.01), headache (P<0.01), and PGE2 (P<0.01). No effect of oxygen was found on PGD2, PGF2, PGI2, TXA2. Placebo oxygen administration on day 3 at 8pm produced the same effects as those of real oxygen. In fact, although SO2 did not change, placebo administration induced decreases in Vmin, pH, headache, PGE2 (P<0.01 by SNK).

**Fig 5:**
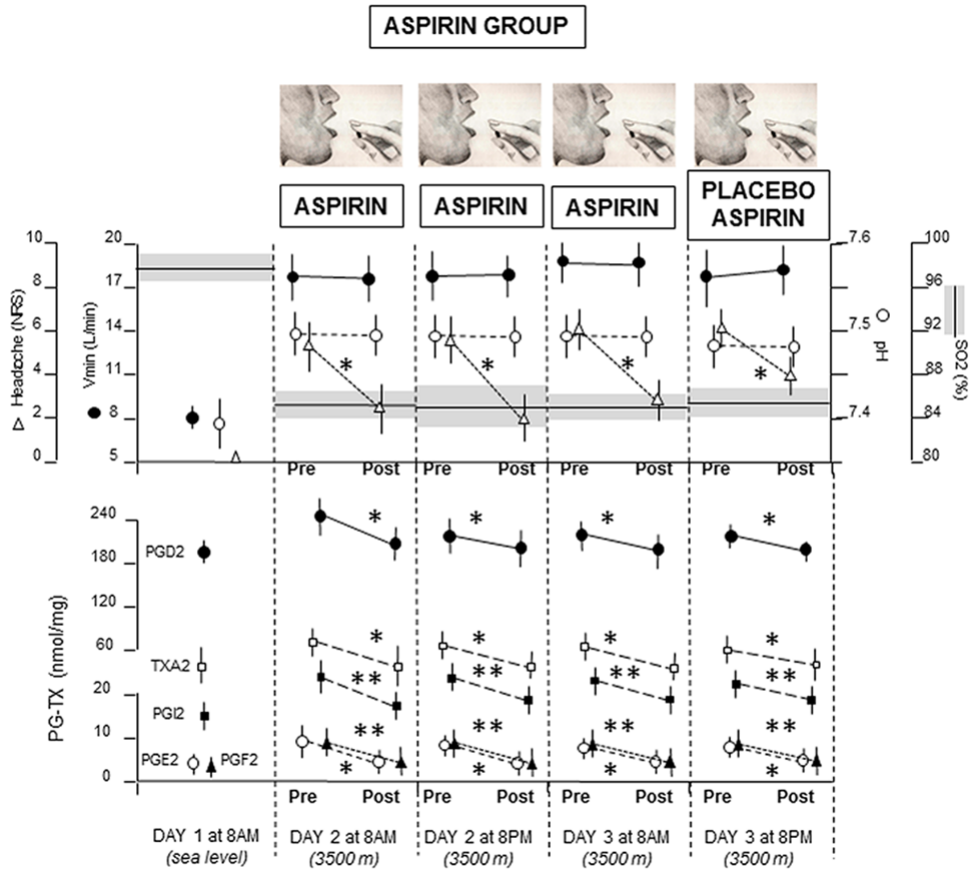
Means (+SD) for all the measurements in the aspirin group. After aspirin administration for three consecutive sessions, real aspirin was replaced with placebo aspirin. Note that placebo mimicked the effects of real aspirin for headache pain, PGE2, PGD2, PGF2, PGI2, TXA2, but it had no effect on SO2, Vmin, pH. *P<0.01; **P<0.05.

In the Aspirin group (Fig 5), SO2 decreased at high altitude whereas all other parameters increased. Aspirin administration on day 2 and 3 neither increased SO2 nor decreased Vmin and pH, but it had a significant effect on headache (P<0.01 by SNK), PGD2 (P<0.01), PGE2 (P<0.01, PGF2 (P<0.05), PGI2 (P<0.05), TXA2 (P<0.01). Placebo aspirin administration on day 3 at 8pm produced the same effects as those of real aspirin. In fact, whereas SO2, Vmin, pH did not change after placebo, headache, PGD2, PGE2, PGF2, PGI2, TXA2 decreased (P<0.01 for all).

**Fig 6:**
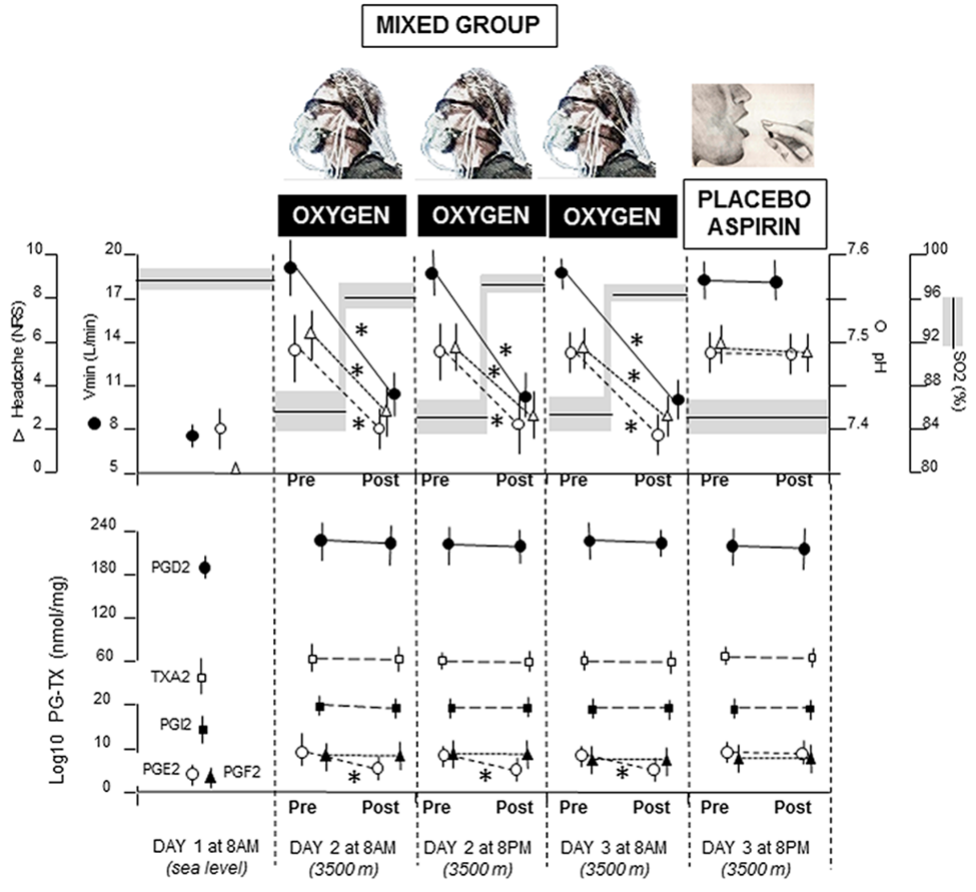
Means (+SD) for all the measurements in the mixed group. After oxygen administration for three consecutive sessions, real oxygen was replaced with placebo aspirin. Note that none of the parameters was affected by placebo aspirin. *P<0.01.

Overall, Figs 4 and 5 show that placebos mimic the effect of the previously administered treatment. Whereas placebo oxygen affects the Vmin-pH pathway by decreasing hyperventilation and alkalosis, along with PGE2, placebo aspirin reduces all the cyclooxygenase products. Large effect sizes, calculated by means of Cohen’s *d*, were found in the placebo oxygen group (Fig 4) for Vmin (*d* = 2.8), pH (*d* = 4.12), pain (*d* = 4.32), PGE2 (*d* = 1.51), and in the aspirin placebo group (Fig 5) for pain (*d* = 2.4), PGD2 (*d* = 2.98), PGE2 (*d* = 2.69), PGF2 (*d* = 1.8), PGI2 (*d* = 2.44), TXA2 (*d* = 1.58). In Fig 6 it can be seen that placebo aspirin had no effect if pre-conditioning was carried out with oxygen. In fact, oxygen administration induced a decrease of both Vmin and pH, as well as PGE2, but this effect could not me mimicked by placebo aspirin, which indicates high specificity in the conditioning procedure and a possible direct effect of alkalosis on PGE2, without the intervention of cyclooxygenase.

### Between-groups analysis

In order to further quantify these effects, we performed an inter-groups analysis to better identify the differences between the two placebos. To do this, we computed the differences of the means between placebo oxygen (group 2) and no-treatment (group 1) in order to identify the placebo oxygen effect, the differences of the means between placebo aspirin (group 3) and no-treatment (group 1) in order to identify the placebo aspirin effect, the differences of the means between placebo oxygen (group 2) and placebo aspirin (group 3) in order to identify the differences between the two placebos, and the differences of the means between placebo aspirin after oxygen conditioning (group 4) and no-treatment (group 1) in order to assess the placebo aspirin effect after oxygen prior conditioning.

**Fig 7:**
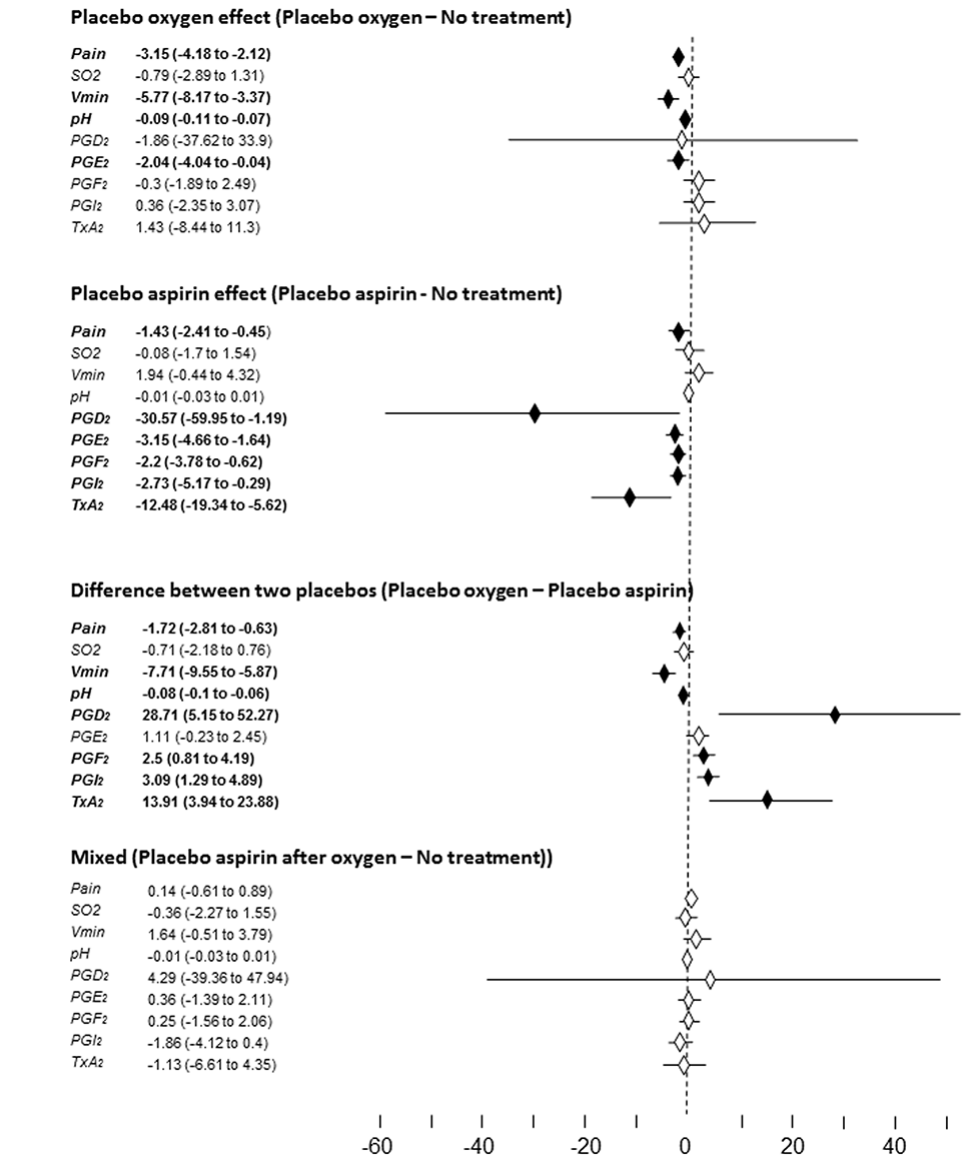
Differences of the means and 95% CI for all the parameters analyzed in the present study. Horizontal bars crossing the 0 line and white diamonds are statistically nonsignificant. Horizontal bars not crossing the 0 line and black diamonds are significant. From top to bottom: i) placebo oxygen effect is represented by the difference between placebo oxygen an no-treatment, ii) placebo aspirin effect is the difference between placebo aspirin and no-treatment, iii) the difference between the two placebos is the difference between placebo oxygen and placebo aspirin, iv) the mixed effect is represented by the difference between placebo aspirin after oxygen conditioning and no-treatment.

The detailed statistical comparisons with the differences of the means and the 95% CI are shown in Fig 7. As to the differences of the means between placebo oxygen and no-treatment (placebo oxygen effect), it can be seen that, whereas there was a significant placebo oxygen effect for pain, Vmin, pH, and PGE2, no placebo effect was present for SO2, PGD2, PGF2, PGI2, and TXA2. By contrast, the comparison between placebo aspirin and no-treatment showed a significant placebo aspirin effect for pain, PGD2, PGE2, PGF2, PGI2, TXA2, but not for SO2, Vmin, pH. Statistical comparison between placebo oxygen and placebo aspirin showed significant effects for pain, Vmin, pH, PGD2, PGF2, PGI2, and TXA2, but not for SO2 and PGE2. Therefore, the two placebos produced completely different outcomes, with the exception of SO2 and PGE2. No significant differences were found when comparing placebo aspirin after oxygen pre-conditioning and no-treatment.

## Discussion

**Fig 8:**
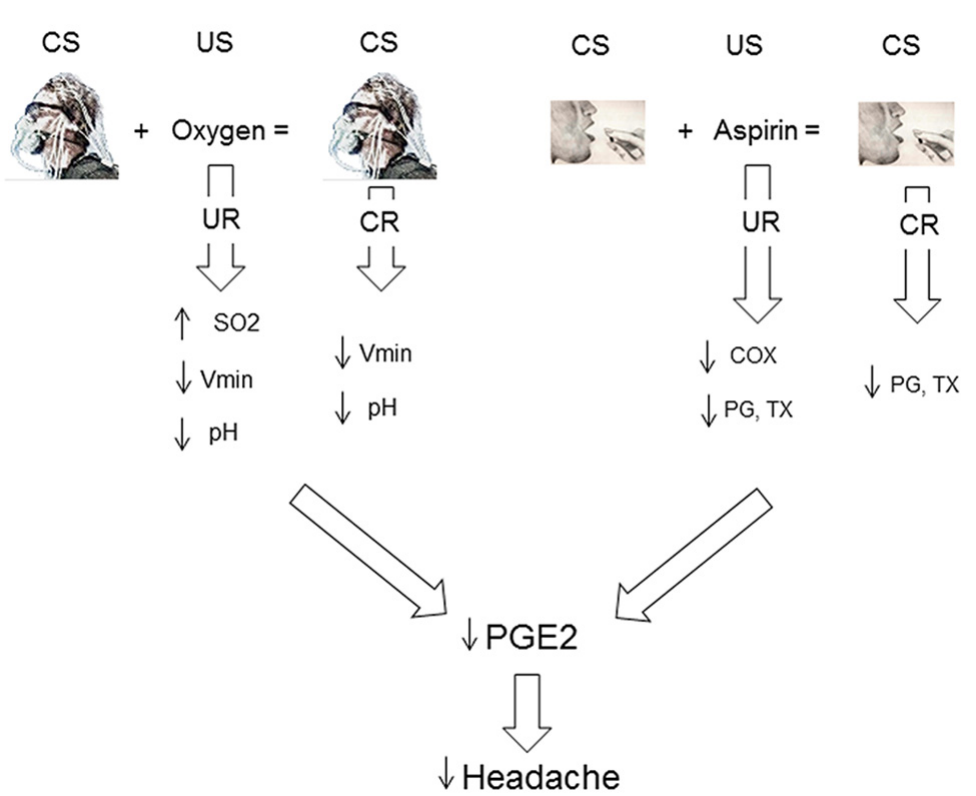
Model that explains the findings of the present study. The repeated association between a conditioned stimulus (CS) and an unconditioned stimulus (US) leads to a conditioned response (CR), in which the CS alone (the placebo) is capable of inducing the same unconditioned response (UR) of the US. In the case of oxygen treatment, the CS (the ritual of the oxygen mask) induces a CR characterized by a decrease in Vmin and pH. In the case of aspirin treatment, the CS (the ritual of the pill) induces a CR characterized by a decrease in prostaglandins (PG) and thromboxane (TX). The present study suggests that PGE2 might be the final common pathway of the analgesic effect. In fact, both placebos decrease PGE2.

The findings of this study suggests the model depicted in Fig 8. The ritual of the oxygen mask, acting as a conditioned stimulus (CS), associated several times with oxygen delivery, acting as the unconditioned stimulus (US), leads to a conditioned response (CR), whereby the mask ritual alone is capable of inducing the same unconditioned response (UR) of the oxygen, yet without any increase in SO2. Likewise, the ritual of the aspirin pill (CS), associated several times with aspirin delivery (US), leads to a CR, whereby the pill ritual alone is capable of inducing the same UR of the aspirin. Thus, these two placebos trigger different mechanisms. The mask ritual induces a decrease in Vmin and pH, whereas the pill ritual induces a decrease in PGs and TX. In other words, the two placebos mimic the effect of the treatment which the ritual was paired to. The present study also suggests that PGE2 reduction might be the final common pathway of the analgesic effect of both the mask and the pill ritual. In fact, in both situations we found a decrease in PGE2.

The high specificity of the mechanisms activated by the two placebos is shown by the mixed group, in which the administration of placebo aspirin after oxygen pre-conditioning was totally ineffective. In other words, if the mask ritual CS is replaced with a different CS (the pill ritual), the placebo pill CS does not induce any CR. This specificity resembles the high specificity of the pharmacological pre-conditioning with either opioid or nonopioid drugs. If morphine is given for two consecutive days and then replaced with a placebo, the placebo response is mediated by the opioid receptors, but if the pre-conditioning is performed with nonopioid drugs, such as ketorolac, the placebo response is mediated by the cannabinoid receptors [23,24].

High altitude headache is a complex clinical condition with a multifactorial pathophysiology. For example, an important mechanism of the acute effects of hypoxia on headache pain is represented by the increase in PG synthesis through the cyclooxygenase enzyme [7,11]. One of the most important effects of PGs, particularly PGE2, is represented by vasodilation, which is thought to be the principal factor inducing acute hypoxia headache [12–16]. In addition, the direct stimulation of nociceptive afferents by PGE2 may also take place [17]. However, this mechanism does not seem to operate alone. A second factor that is involved in high altitude headache is alkalosis, which is due to the hyperventilation compensatory response with the subsequent excessive elimination of CO2 [18]. Indeed, acidification of the blood by means of drugs such as acetazolamide is effective in preventing headache pain [19].

Changes in pH have been found to modulate endothelium-dependent contractions in mouse arteries by altering the sensitivity of thromboxane/prostanoid receptors of vascular smooth muscles to endothelium-derived contracting factors [25]. Moreover, vasodilation has been found to be induced by alkalosis, for example in the piglet pulmonary vessels [26,27]. Interestingly, hyperventilation stimulates the release of PGE2 and PGI2 from lungs in humans [28]. Since the oxygen-induced reduction in PGE2 of the present study was not mimicked by placebo aspirin (Fig 6), this suggests that alkalosis might act on PGE2 directly, without affecting cyclooxygenase activity. Indeed, all the other products of cyclooxygenase did not change after oxygen administration. Unfortunately, our study cannot be conclusive in this sense, and further research should be aimed at understanding the relationship between hyperventilation/alkalosis and PGE2 in cerebral vessels in order to better define the pathophysiology of hypoxia-induced headache.

The fact that all placebos are not equal, as previously found in a number of conditions, such as migraine [1] and osteoarthritis [2] leads to important theoretical and practical implications. For example, Kong et al [5] found that individuals may respond to unique healing rituals in different ways and in different situations, e.g. to pills or acupuncture needles, and this could explain the difficulty of detecting a signature for placebo responders. In other words, the placebo response may be a complex behavioral phenomenon that is associated to a state rather than a trait characteristic. This may have profound implications for clinical trial designs and should be explored further in future research.

It is worth noting that previous studies on the placebo response in high altitude headache showed that placebos are effective only after a pre-conditioning procedure [6,7]. The present study was devised on the basis of these previous findings. Therefore, we induced placebo responses to either placebo oxygen or placebo aspirin following prior exposure to either oxygen or aspirin, respectively. This further highlights the limits of a placebo treatment in this medical condition, in which only a learning procedure is capable of producing significant placebo analgesic responses. It should also be noted that in our previous study we found that placebo may induce an analgesic effect in high altitude headache, yet without affecting SO2, an apparently surprising finding [6]. Indeed, how can headache pain be reduced if the hypoxic state, and thus low SO2, persists? As shown in the present study, placebo oxygen acts on Vmin and blood pH rather than on SO2 per se. Therefore, repeated administrations of real oxygen produce hypoventilation responses which, in turn, lead to a conditioned hypoventilation.

The hypoventilation response is in agreement with previous studies which showed a placebo respiratory depression after pre-conditioning with the narcotic drug, buprenorphine [29,30]. If buprenorphine is given for several days in a row to induce respiratory depression, and then it is replaced with a placebo, the placebo mimics the respiratory depressant effects of the previously administered buprenorphine. Therefore, conditioned placebo hypoventilation can be induced by both oxygen in hypoxic conditions and by narcotic drugs.

Some limitations of our study need to be discussed. First, due to the unusual situation at high altitude, the number of the subjects/group was necessarily limited. However, it should be noted that high altitude headache is an interesting model to understand some of the neurobiological placebo mechanisms because it involves physiological parameters that have not been investigated in placebo research so far. Furthermore, the therapeutic approach with different procedures allows us to investigate the clinical and biological effects of different rituals. A second limitation that needs to be acknowledged is that some pathophysiological mechanisms of high altitude headache are still unclear, thus the mechanisms we have described in the present study may represent only a part of a more complex picture that needs to be unraveled. A third limitation could be related to oxygen delivery, which in our case was at low flow rate (7 L/min). Since previous studies found a small placebo effect in cluster headache in a trial with low oxygen flow rate (6 L/min) [31] compared to a trial with high flow (12 L/min) [32], future research should aim to verify whether flow rate could be a crucial parameter for inducing substantial placebo responses. A fourth limitation is that we do not know what would happen if the placebo was given without pre-conditioning. However, it should be noted that previous studies showed no placebo response without prior exposure to oxygen [6], and indeed the present study was conceived on this ground.

Our findings underscore the importance of using the appropriate placebos and outcome measures in clinical trials. Since all placebos are not equal and since they may trigger different mechanisms, the choice of inappropriate primary and secondary outcome measures may be crucial in the interpretation of the data. For example, in our study, outcome measures such as Vmin and pH were affected by one type of placebo but not by the other. Modern clinical trials should consider all these findings in order to better identify and isolate placebo effects. Although the task is surely difficult and challenging, the only way to do this is to understand the mechanisms of different placebos and rituals across all medical conditions and therapeutic interventions.

## Supporting Information

S1 Table Raw data for all groups. (XLS)
